# Targeted amplicon sequencing + next-generation sequencing–based bulked segregant analysis identified genetic loci associated with preharvest sprouting tolerance in common buckwheat (*Fagopyrum esculentum*)

**DOI:** 10.1186/s12870-020-02790-w

**Published:** 2021-01-06

**Authors:** Ryoma Takeshima, Eri Ogiso-Tanaka, Yasuo Yasui, Katsuhiro Matsui

**Affiliations:** 1 Institute of Crop Science, National Agriculture and Food Research Organization (NARO), Kannondai 3-1-3, Tsukuba, Ibaraki, 305-8518 Japan; 2 Graduate School of Agriculture, Kyoto University, Kitasirakawa Oiwake-Cho, Sakyou-ku, Kyoto, 606-8501 Japan; 3 Graduate School of Life and Environmental Science, University of Tsukuba, Kannondai 3-1-3, Tsukuba, Ibaraki, 305-8518 Japan

**Keywords:** Breeding, Genetic maps, Genome database, Resequencing, Marker-assisted selection, QTL-Seq

## Abstract

**Background:**

Common buckwheat (2*n =* 2*x =* 16) is an outcrossing pseudocereal whose seeds contain abundant nutrients and potential antioxidants. As these beneficial compounds are damaged by preharvest sprouting (PHS) and PHS is likely to increase with global warming, it is important to find efficient ways to develop new PHS-tolerant lines. However, genetic loci and selection markers associated with PHS in buckwheat have not been reported.

**Results:**

By next-generation sequencing (NGS) of whole-genome of parental lines, we developed a genome-wide set of 300 markers. By NGS- based bulked segregant analysis (NGS-BSA), we developed 100 markers linked to PHS tolerance. To confirm the effectiveness of marker development from NGS-BSA data, we developed 100 markers linked to the self-compatibility (SC) trait from previous NGS-BSA data. Using these markers, we developed genetic maps with AmpliSeq technology, which can quickly detect polymorphisms by amplicon-based multiplex targeted NGS, and performed quantitative trait locus (QTL) analysis for PHS tolerance in combination with NGS-BSA. QTL analysis detected two major and two minor QTLs for PHS tolerance in a segregating population developed from a cross between the PHS-tolerant ‘Kyukei 29’ and the self-compatible susceptible ‘Kyukei SC7’. We found different major and minor QTLs in other segregating populations developed from the PHS-tolerant lines ‘Kyukei 28’ and ‘NARO-FE-1’. Candidate markers linked to PHS developed by NGS-BSA were located near these QTL regions. We also investigated the effectiveness of markers linked to these QTLs for selection of PHS-tolerant lines among other segregating populations.

**Conclusions:**

We efficiently developed genetic maps using a method combined with AmpliSeq technology and NGS-BSA, and detected QTLs associated with preharvest sprouting tolerance in common buckwheat. This is the first report to identify QTLs for PHS tolerance in buckwheat. Our marker development system will accelerate genetic research and breeding in common buckwheat.

**Supplementary Information:**

The online version contains supplementary material available at 10.1186/s12870-020-02790-w.

## Background

Common buckwheat (*Fagopyrum esculentum* Moench; 2*n =* 2*x =* 16) is an outcrossing pseudocereal owing to heterostylous self-incompatibility (SI). It is widely grown in the temperate zones of the world. Buckwheat seeds contain health-promoting compounds with antioxidative, antihypertensive, and anti-obesity properties [1, 2], in addition to high levels of starch and high-quality protein with a well balanced amino acid profile [3, 4]. However, these beneficial compounds are strongly influenced by external effects; in particular, germination on the plant, called preharvest sprouting (PHS), severely degrades seed quality.

**Fig. 1:**
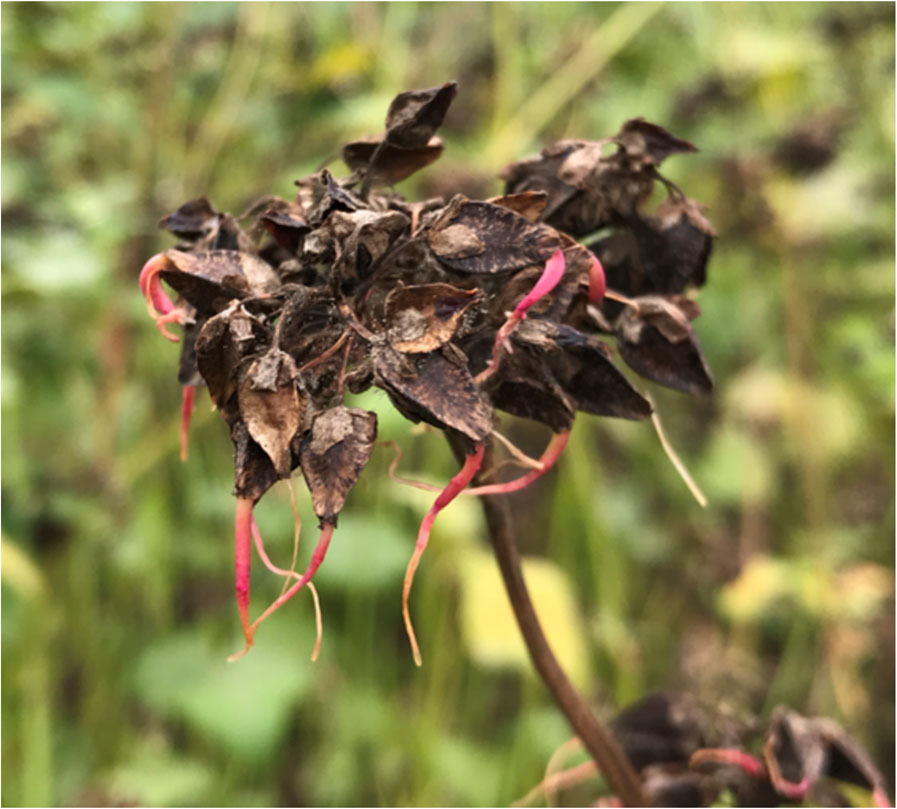
Preharvest sprouting of common buckwheat. This photo was originally taken at a buckwheat breeding field in the Institute of Crop Science, NARO

PHS often occurs under the humid and warm conditions common before harvest (Fig. 1). It degrades the pasting viscosity and quality of buckwheat flour, and decreases the total content of starch and crude fat [5–7]. Global warming might extend the range where PHS occurs [8]. Therefore, improving PHS tolerance of buckwheat is a major breeding target worldwide.

We developed four PHS-tolerant cultivars/breeding lines—‘Harunoibuki’, ‘NARO-FE-1’ (NF1), ‘Kyukei 28’ (KY28), and ‘Kyukei 29’ (KY29)—by mass selection of low-PHS individuals [8, 9]. To clarify the inheritance of the PHS tolerance of these lines, we performed genetic analysis using segregating populations derived from crosses between KY28 or KY29 and the self-compatible PHS-susceptible ‘Kyukei SC7’ (KSC7) [8]; KSC7 was developed by the introduction of the self-compatibility (SC) allele of a wild relative, *F*. *homotropicum* [10]. In the F_2_ progeny derived from KY28 × KSC7, the segregation pattern of the frequency of PHS suggested that several recessive genes regulate PHS tolerance in KY28. On the other hand, in the F_2_ progeny derived from KY29 × KSC7, the segregation pattern fitted the expected ratio of two dominant genes (15:1), suggesting that the PHS tolerance of KY29 is controlled by two major genes [8]. Because buckwheat is an outcrossing plant, it is difficult to fix favorable traits such as PHS tolerance. Although marker-assisted selection (MAS) is an efficient way to do so [10], quantitative trait locus (QTL) and selection markers for PHS tolerance in buckwheat have not been reported until now.

Recently, we developed the buckwheat genome database (BGDB, http://buckwheat.kazusa.or.jp/) [11] to support genetic analysis and marker development in buckwheat [12]. We developed codominant markers linked to the region flanking the gene for self-incompatibility/compatibility (SI/SC) by next-generation sequencing (NGS)-based bulked-segregant analysis (BSA) in F_2_ progeny [13]. QTL-seq, which is used for whole-genome resequencing (WGS) with BSA, is a powerful tool for the rapid detection of QTLs [14]. However, it needs a database of long scaffolds from which physical maps can be drawn. Unfortunately, the usable reference sequence in BGDB are short (N50 = 25.1 kb) [11], so it is necessary to construct a genetic linkage map for detecting QTLs.

To construct a genetic linkage map efficiently, we need a genome-wide marker set and an efficient genotyping system. The Ion AmpliSeq Targeted Sequencing technology (Thermo Fisher Scientific, Waltham, MA, USA) can quickly detect polymorphisms by amplicon-based multiplex targeted NGS [15, 16]. Here, we developed genetic maps with AmpliSeq and sought QTLs for PHS tolerance in buckwheat by NGS-BSA. We developed genome-wide markers for QTL analysis and detected several QTLs related to PHS tolerance. In addition, we developed linked markers and investigated the effect of selection with the markers. Furthermore, we demonstrated the effectiveness of NGS-BSA by developing linkage maps from AmpliSeq data of markers linked to the SC allele. Our findings and marker development system will be useful for advancing genetic research for buckwheat breeding.

## Results

### Distribution frequencies of PHS in three populations

**Fig. 2:**
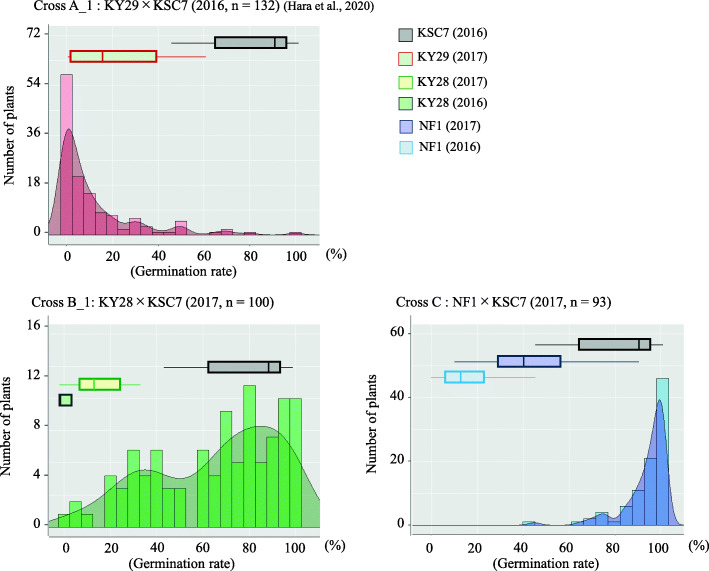
Segregation of germination rates in progeny of three crosses. **a**_1, Kyukei 29 (KY29) × Kyukei SC7 (KSC7) [8]. **b**_1, Kyukei 28 (KY28) × KSC7. **c** NARO-FE-1 (NF1) × KSC7. Histograms, numbers of plants; curves, kernel density estimates; box plots, germination rates of parents

We investigated the distribution frequencies of PHS tolerance in parental lines and five F_2_ populations developed by self-pollination of each F_1_ plant derived from crosses between the highly PHS-tolerant lines KY29, KY28, and NF1 and the PHS-susceptible SC line KSC7 (Fig. 2; Additional files 1 and 2: Table S1, S2). The F_2_ populations derived from KY29 × KSC7 (A_1, A_2) and from KY28 × KSC7 (B_2) are already reported [8]. We investigated those derived from KY28 × KSC7 (B_1) and NF1 × KSC7 (C) here (Additional file 1: Table S1). High rates of PHS tolerance in the progeny of cross A_1 suggest that major dominant tolerance genes in KY29 are involved (Fig. 2) [8]. Low rates of tolerance in the progeny of cross B_1 suggest that major recessive tolerance genes in KY28 are involved (Fig. 2) [8]. To see whether the newly developed PHS-tolerant line NF1 has a different pattern of inheritance, we developed cross C. Its F_2_ progeny showed mostly low tolerance to PHS, suggesting that major recessive tolerance genes in NF1 are involved, as in KY28 (Fig. 2). However, to determine which other genes are related to PHS tolerance in each population, QTL analysis is needed.

### Development of marker sets to cover the whole genome and linkage markers

To develop genome-wide AmpliSeq markers, we first performed local BLAST searches of all microarray probe sequences of Yabe et al. [17] with BGDB genome data to find scaffolds with the matching sequences. Among all 1129 probe sequences, 1063 probes matched 387 scaffolds distributed in eight linkage groups (Additional file 3: Table S3). We used these 387 scaffolds as genome-wide scaffolds. From these, we selected 300 SNP sites as genome-wide SNPs for the AmpliSeq analysis (Additional file 4: Table S4).

To develop the PHS-linked marker set, we compared sequences between a high-PHS-tolerance bulk and a low-PHS-tolerance bulk of DNA of plants in F_2_ population of KY29 × KSC7 (A_1) and obtained 535 scaffolds with high values of the PHS-linked SNP index (≥0.700; Additional file 5: Table S5). Here, we selected 100 scaffolds and investigated the linkage relations.

To investigate the effect and certainty of NGS-BSA, we made an AmpliSeq marker set that is linked to the SC trait, *S*^*h*^. We had already performed NGS-BSA at the locus and developed codominant markers, but we used only the top 50 candidate scaffolds [13]. Here, we selected 100 scaffolds with high SNP-index in previously published WGS data of KSC7 (LH) and KY29 (pin) [13] and investigated the linkage relations.

Finally, we developed a custom panel of 500 markers which amplify 100 PHS-linked SNPs, 100 *S*^*h*^-linked SNPs, and 300 genome-wide SNPs (Additional file 6: Table S6).

### Construction of linkage maps by AmpliSeq in three F_2_ populations

**Fig. 3:**
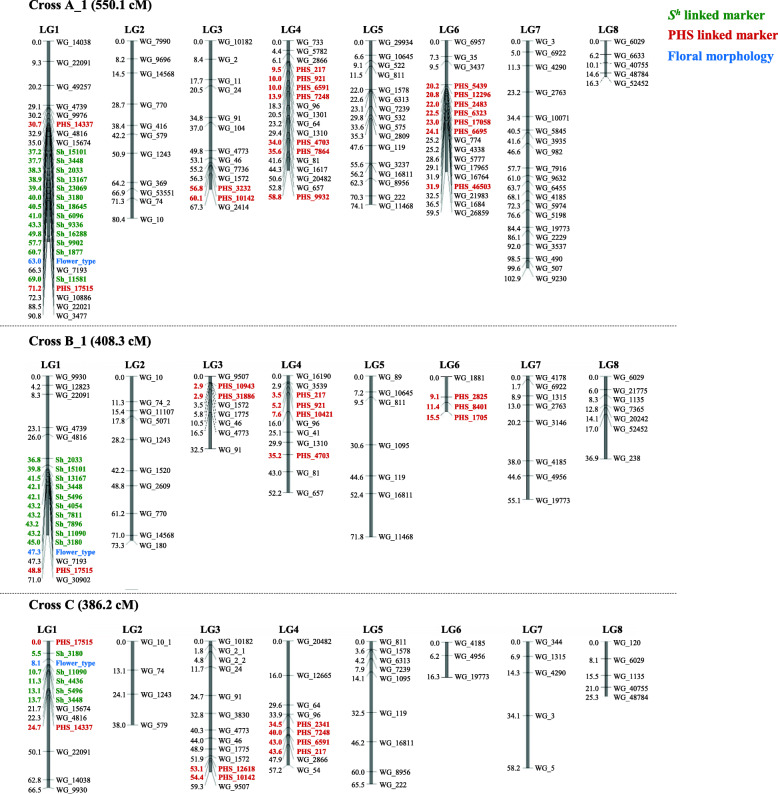
Genetic maps of crosses **a**_1 (KY29 × KSC7), **b**_1 (KY28 × KSC7), and **c** (NF1 × KSC7)

Using this 500-marker set, we genotyped progeny derived from crosses A_1, B_1, and C by AmpliSeq. All genotyping data were filtered by R/qtl and linkage maps were constructed (Fig. 3). Unfortunately, several of the markers did not show SNPs and so could not be used for mapping. This may be because the BGDB reference genome contains a large number of unidentified nucleotide sequences ‘N’, which prevented elimination of the off-target hybridization in Ion AmpliSeq Designer (Additional file 10: Fig. S1). All detected polymorphisms and usable markers are summarized in Table 1. All three populations gave eight linkage groups (LGs) with a total map length of 550.1 cM in A_1, 408.3 cM in B_1, and 386.2 cM in C (Fig. 3).

**Table 1:** AmpliSeq results and classification of markers

	Cross A_1 (*n* = 94)				Cross B_1 (*n* = 87)				Cross C (*n* = 84)			
	***S***^***h***^ link^**a**^	PHS link^**a**^	Whole-genome^**a**^	Total	***S***^***h***^ link	PHS link	Whole-genome	Total	***S***^***h***^ link	PHS link	Whole-genome	Total
Number of SNP detected markers	67	56	176	299	74	41	124	239	68	34	140	242
After filtering and before removing duplicate^b^	57	48	112	217	50	14	61	125	43	11	55	109
Remove duplicated markers (no. of duplicated marker)	12 (45)	18 (30)	98 (14)	127 (90)	10 (40)	10 (4)	54 (7)	74 (51)	5 (38)	8 (3)	49 (6)	62 (47)
LG1	12 (45)	2 (1)	13 (2)	27 (48)	10 (40)	1 (1)	8 (3)	19 (44)	5 (38)	2 (1)	6 (3)	13 (42)
LG2	0	0	11 (3)	11 (3)	0	0	10	10	0	0	4 (1)	4 (1)
LG3	0	2 (3)	11 (4)	13 (7)	0	2 (2)	6	8 (2)	0	2 (1)	11	13 (1)
LG4	0	7 (1)	11 (3)	18 (4)	0	4 (1)	7 (1)	11 (2)	0	4 (1)	6	10 (1)
LG5	0	0	15	15	0	0	7	7	0	0	9	9
LG6	0	7 (25)	11 (1)	18 (26)	0	3	1	4	0	0	3 (1)	3 (1)
LG7	0	0	20	20	0	0	8 (1)	8 (1)	0	0	5	5
LG8	0	0	5 (1)	5 (1)	0	0	7 (2)	7 (2)	0	0	5 (1)	5 (1)

^a^Expected marker linkage: *S*^*h*^-link, self-compatibility; PHS link, tolerance to preharvest sprouting; whole-genome, whole genome region

^b^Filtering steps: drop duplicate markers; drop samples with < 80 genotypes; drop marker subsets in which < 90% of population is genotyped; drop markers with an abnormal genotyping distribution

The flower morphology (LH/pin) marker, labeled “Flower_type” on the maps, was located on LG1 (Fig. 3). As expected, most *S*^*h*^-linked markers developed by NGS-BSA were mapped around it in all populations. As we omitted 45 *S*^*h*^-linked markers as duplicates in cross A_1, 40 in B_1, and 38 in C during filtering (Table 1), the resultant *S*^*h*^-linked markers were tightly linked to the *S*^*h*^ locus, indicating the efficiency and accuracy of NGS-BSA for a trait in buckwheat controlled by a single major gene.

The results of mapping with all markers and the *S*^*h*^-linked markers suggest that the AmpliSeq marker development system functioned well and can be used for PHS analysis. Two-thirds of PHS-linked markers (32/48) were clustered and mapped on LG6 in A_1, and several markers were mapped separately on LG1, LG3, and LG4 in each population (Fig. 3; Table 1). Furthermore, the deletion of 25 of 32 PHS-linked markers as duplicates on LG6 in cross A during filtering suggests that the main genetic locus controlling the PHS tolerance of KY29 could be located on LG6.

### QTL analysis for PHS tolerance

**Fig. 4:**
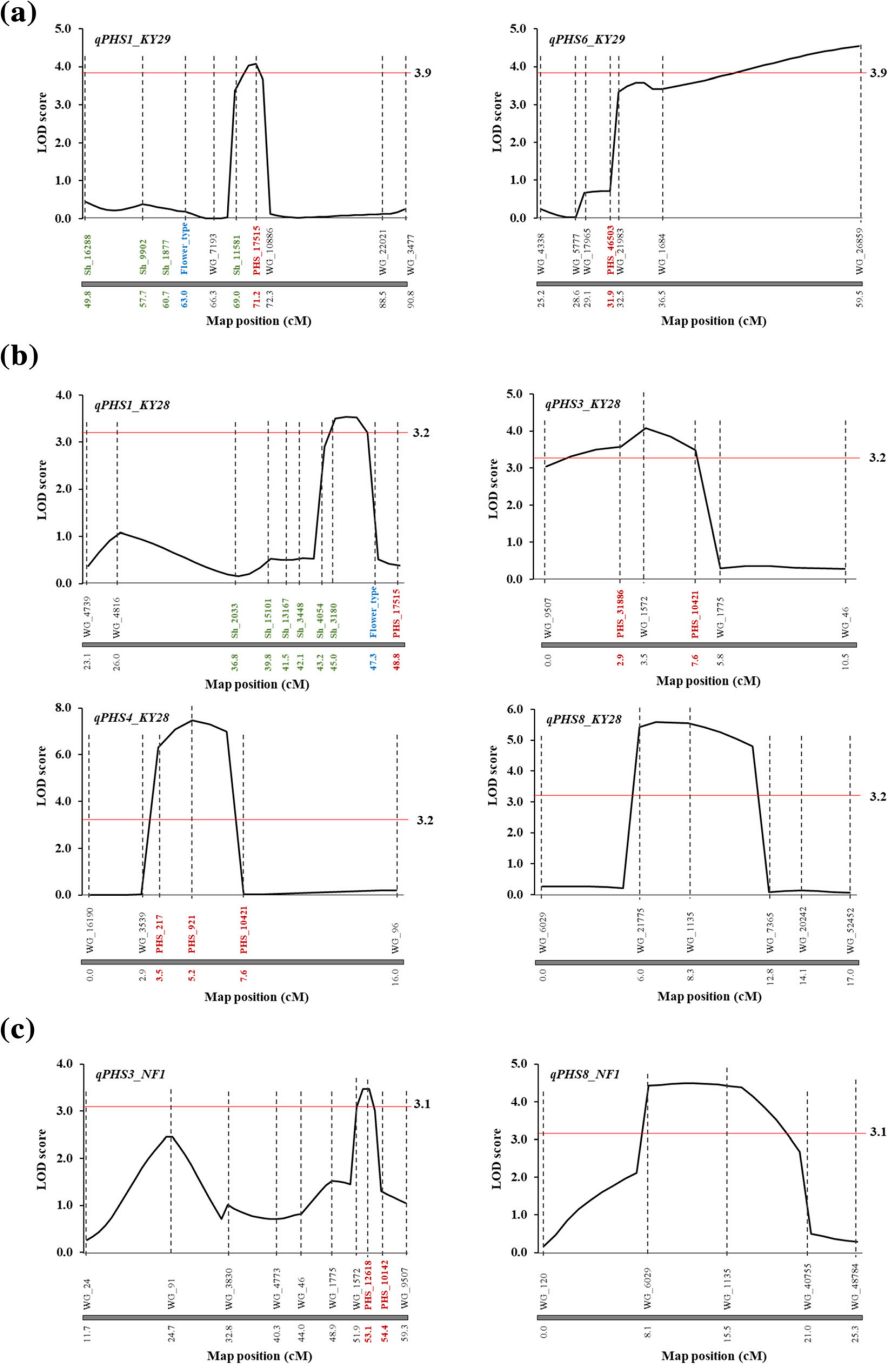
QTLs detected in each cross: **a**, Cross A_1; **b**, Cross B_1; **c**, Cross C

We performed QTL analysis for PHS tolerance by composite interval mapping (CIM). The thresholds of log-likelihood (LOD) significance (*P* < 0.05) were 3.9 in A_1, 3.2 in B_1, and 3.1 in C. In A_1, two major and two minor QTLs (LOD threshold ± ≥2.0) were detected (Table 2, Fig. 4), and explained 42.2% of phenotypic variation (Table 2). QTLs *qPHS1_KY29*, *qPHS6_KY29*, and *qPHS4_KY29* confer tolerance with the KY29 genotype, and *qPHS7_KY29* confers tolerance with the KSC7 genotype. *qPHS6_KY29* had the largest effect (15.6%) and was strongly dominant. In B_1, four major and one minor QTLs were detected (Table 2, Fig. 4), and explained 66.4% of phenotypic variation. QTLs *qPHS3_KY28* and *qPHS4_KY28* confer tolerance with the KY28 genotype, and *qPHS1_KY28*, *qPHS5_KY28*, and *qPHS8_KY28* confer tolerance with the KSC7 genotype. *qPHS4_KY28* had the largest effect (22.0%) and appeared to be recessive. In C, two major and one minor QTLs were detected (Table 2, Fig. 4), and explained 38.0% of phenotypic variation. QTLs *qPHS2_NF1* and *qPHS3_NF1* confer tolerance with the NF1 genotype, and *qPHS8_NF1* confers tolerance with the KSC7 genotype. *qPHS3_NF1* and *qPHS8_NF1* are located near the marker regions (WG_1572 and WG_1135) for *qPHS3_KY28* and *qPHS8_KY28* (Table 2).

**Table 2:** QTLs for preharvest sprouting

Populations	QTLs	LG	Closest marker	peak position (cM)	LOD	Additive effect^**b**^	Dominant effect	R2 (%)^**c**^
			(position (cM))					
Cross A_1	*qPHS1_KY29*	1	Sh_11581 –PHS_17515	71.2	4.08	10.29	−0.48	11.7
			(69.0)–(71.2)					
	*qPHS6_KY29*	6	WG_1684 – WG_26859	59.5	4.54	12.21	−7.44	15.6
			(36.5)–(59.5)					
	*qPHS4_KY29*^a^	4	WG_657 – PHS_9932	57.8	2.44	8.26	−3.5	7.4
			(52.8)–(58.8)					
	*qPHS7_KY29*^a^	7	WG_982 – WG_7916	49.6	2.35	−7.05	−3.3	7.5
			(46.6)–(57.7)					
Cross B_1	*qPHS1_KY28*	1	Sh_11090 – Sh_3180	45	3.54	−9.18	6.45	9
			(43.2)–(45.0)					
	*qPHS3_KY28*	3	PHS_31886 – WG_1572	3.5	4.09	12.78	5.61	10.6
			(2.9)–(3.5)					
	*qPHS4_KY28*	4	PHS_921 – PHS_10421	5.2	7.48	17.28	−0.36	22
			(5.2)–(7.6)					
	*qPHS8_KY28*	8	WG_21775 – WG_1135	7	5.58	−14.83	−1.84	15.7
			(6.0)–(8.3)					
	*qPHS5_KY28*^a^	5	WG_119 – WG_16811	47.6	2.82	−5.91	11.62	7.1
			(44.6)–(52.4)					
Cross C	*qPHS3_NF1*	3	WG_1572 – PHS_12618	53.2	3.47	4.44	4.26	12
			(51.9)–(53.2)					
	*qPHS8_NF1*	8	WG_6029 –WG_1135	12.1	4.49	−4.16	4.64	16
			(8.1)–(15.5)					
	*qPHS2_NF1*^a^	2	WG_74 – WG_1243	24.1	3.06	3.58	4.35	10
			(13.1)–(24.1)					

^a^Minor QTLs

^b^Effect contributed by KSC7 alleles

^c^Percentage of total variation in marker association for each trait across population explained by QTL

Our PHS-linked markers in F_2_ progeny of A_1 were located on LGs 1, 3, 4, and 6 (Table 1). QTLs which provide PHS tolerance with the KY29 genotype were detected near these markers (Fig. 2, Table 2), except on LG3. In addition, many PHS-linked markers were clustered and mapped on LG6, and the QTL with the largest effect was detected on LG6 (*qPHS6_KY29*; Table 2) in A_1. These results show that the PHS-linked markers developed by NGS-BSA effectively detected genetic regions for PHS tolerance.

### Confirmation of the effect of QTLs on PHS tolerance by development of sequence-tagged-site markers

To confirm the association between the major QTLs and germination rate, we converted the markers nearest to sequence-tagged-site (STS) markers and investigated the relations between their genotype and the germination rate in segregating populations of crosses A_1, A_2, B_1, and B_2 (Table 3). The markers nearest to *qPHS1_KY29* (LG1) and *qPHS6_KY29* (LG6) were PHS_17515 and WG_26859 (Fig. 3, Table 2); they were converted to STS markers *qPHS1_KY29_PHS_17515* and *qPHS6_KY29_WG_26859* (Additional file 7: Table S7). Alleles of each marker derived from KSC7 and KY29 were designated as A and B, respectively (Table 3). In A_1, plants homozygous for *qPHS1_KY29_B* and *qPHS6_KY29_B* had a lower germination rate than plants homozygous for *qPHS1_KY29_A* and *qPHS6_KY29_A* (Table 3). Although the effects in A_2 were not significant, the effect was similar (Table 3). In A_1, the average germination rate of plants heterozygous for *qPHS6_KY29* was almost the same as that of plants homozygous for the KY29 allele, suggesting that the KY29 allele at *qPHS6_KY29* was dominant (Table 3). On the other hand, the KY29 allele at *qPHS1_KY29* was partially dominant (Table 3).

**Table 3:** Segregation of genotypes at DNA markers for QTLs and associated rate of preharvest sprouting

Marker name	plant no.	plant number of each genotype			Ratio of pre-harvest sprouting (%)			One-way ANOVA	
Population		AA	AB	BB	AA	AB	BB	F-value	***P***
***qPHS1_KY29_PHS_17515***									
Cross A_1	130	29	69	32	23.3 ± 23.3	10.9 ± 18.1	4.5 ± 7.1	9.14	0.0020 *
Cross A_2	106	21	62	23	11.2 ± 16.4	9.1 ± 12.3	6.2 ± 6.5	0.93	0.4137
***qPHS6_KY29_WG_26859***									
Cross A_1	130	35	59	36	25.5 ± 26.8	8.4 ± 13.4	8.7 ± 12.6	7.13	0.0057 *
Cross A_2	106	20	61	25	9.5 ± 9.6	10.1 ± 13.9	5.4 ± 9.3	1.35	0.2865
***qPHS1_KY28_Sh_3180***									
Cross B_1	100	40	42	18	59.4 ± 30.9	69.0 ± 24.5	69.4 ± 25.5	1.51	0.2483
Cross B_2	106	34	49	23	60.6 ± 28.5	58.8 ± 25.4	55.0 ± 23.2	0.32	0.7309
***qPHS3_KY28_WG_1572***									
Cross B_1	100	27	49	24	77.7 ± 24.7	67.0 ± 25.8	47.7 ± 25.8	9.03	0.0021 *
Cross B_2	106	34	52	20	70.1 ± 21.3	57.5 ± 25.2	41.8 ± 23.0	8.75	0.0024 *
***qPHS4_KY28_PHS_921***									
Cross B_1	100	26	50	24	78.4 ± 21.6	67.4 ± 27.1	46.7 ± 25.0	10.21	0.0012 *
Cross B_2	106	22	59	25	57.0 ± 26.3	61.5 ± 25.6	53.0 ± 26.1	0.99	0.3906
***qPHS8_KY28_WG_21775***									
Cross B_1	100	18	49	33	50.5 ± 30.7	60.7 ± 27.6	80.1 ± 17.8	9.48	0.0017 *
Cross B_2	106	23	57	26	43.5 ± 19.2	57.2 ± 26.8	75.0 ± 19.5	11.01	0.0009 **

A, KSC7 allele; B, KY29 or KY28 allele

*P < 0.05; **P < 0.001

In B_1 and B_2, the markers nearest to *qPHS1_KY28* (LG1), *qPHS3_KY28* (LG3), *qPHS4_KY28* (LG4), and *qPHS8_KY28* (LG8) were converted to STS markers (Additional file 7: Table S7). Plants homozygous for the KY28 alleles at *qPHS3_KY28* (*qPHS3_KY28*_*B*) had a lower germination rate than plants homozygous for the KSC7 alleles (*qPHS3_KY28*_*A*) both in B_1 and B_2. The KY28 alleles at *qPHS4_KY28* (*qPHS4_KY28*_*B*) also had a lower germination rate than plants homozygous for the KSC7 (*qPHS4_KY28*_*A*) in B_1, but not in B_2 (Table 3). On the other hand, plants homozygous for *qPHS8_KY28_B* had a higher germination rate (Table 3). *qPHS3_KY28* and *qPHS8_KY28* decreased germination significantly in B_1 and B_2 (Table 3). In B_1, the average germination rates in plants heterozygous at *qPHS3_KY28* and *qPHS4_KY28* were slightly lower than those in plants homozygous for the KSC7 allele, suggesting that the KY28 alleles at those loci were recessive or partially dominant (Table 3).

## Discussion

### Rapid construction of genetic maps in buckwheat

The development of genome databases makes it easy to do QTL analysis by BSA (QTL-seq) in major crops and model plant species, such as rice, barley, and Arabidopsis [18–20]. By WGS in buckwheat, we developed the BGDB [11], but its small scaffold size so far prevents its use in QTL-seq analysis. In addition, to use genetic maps for QTL analysis, the targeted trait has to segregate in segregating populations. Thus, the construction of genetic maps is still the first step for QTL analysis in buckwheat. However, the construction of maps that cover the whole genome requires much effort, especially in outcrossing plant species.

AmpliSeq technology is often used in cancer research [21] and is beginning to be used in agronomy. Sato et al. [22] developed a highly flexible and repeatable AmpliSeq-based genome-wide genotyping system for aquaculture studies to enhance population genetic studies and genome-wide association study. However, there is no report yet of whole-genome maps constructed using AmpliSeq in crops. Here, we developed a 300-marker genome-wide set by using WGS data of the parents of cross A_1 (KY29 and KSC7) in buckwheat. These markers could detect SNPs not only in A_1, but also in B_1 and C, which were located on 8 LGs in all three populations (Table 1), probably owing to the high nucleotide diversity within cultivated buckwheat (π = 0.0065) [23]. This result suggests that this AmpliSeq-based genome-wide genotyping system allows the efficient and rapid construction of genetic linkage maps in buckwheat.

Furthermore, constructed maps and markers could be useful for QTL analysis of other traits, such as photoperiod sensitivity and flavonoid contents, although the traits should segregate in the segregating population. In addition, increasing the number of markers will be important for fine mapping and more detailed QTL analysis through the comparison of WGS data of SNP/indel information among lines and landraces.

### NGS-BSA + AmpliSeq offers an efficient way to identify QTLs and to develop selection markers in common buckwheat

As the buckwheat draft genome sequence that we have developed is still divided into 387,594 scaffolds, it is impossible to show QTL peaks on a physical map. Here, we used NGS-BSA combined with AmpliSeq to identify QTLs for PHS efficiently and rapidly and to develop tightly linked markers.

The 48 PHS-linked markers developed by NGS-BSA were located on LGs 1, 3, 4, and 6 (Fig. 3, Table 1). Two-thirds of them (32/48) were clustered and mapped on LG6, including one linked to the QTL with the largest effect (*qPHS6_KY29*) in cross A_1 (Tables 1 and 2). Since PHS-linked markers were developed in the F_2_ progeny of cross A_1, we expected that they would be detected in regions derived from KY29 that contribute strongly to PHS resistance. QTLs that provide PHS tolerance with the KY29 genotype were detected on LGs 1 (*qPHS1_KY29*), 4 (*qPHS4_KY29*), and 6 (*qPHS6_KY29*) in cross A_1 (Table 2). On the other hand, a QTL that provides tolerance with the KSC7 genotype was detected on LG7 (*qPHS7_KY29*) (Table 2). Thus, QTL analysis is consistent with the results of NGS-BSA and indicates that AmpliSeq sequencing worked well to detect QTLs for PHS tolerance that originated from KY29. If we had used only NGS-BSA to develop linkage markers, we would not know the linkage relations of these markers and could not select efficient markers, because we could not select markers from each region linked to different QTLs.

We also developed 100 *S*^*h*^-linked markers by NGS-BSA and investigated their genetic regions by mapping them on the same maps as used for PHS QTL analyses. All *S*^*h*^-linked markers were mapped near the region of the floral morphology marker on LG1 in all three populations (Fig. 3, Table 1). This NGS-BSA targeting of one major locus indicates that the method is very effective for developing tightly linked markers. Thus, the combination of NGS-BSA with AmpliSeq is an efficient way to identify genetic regions for both qualitative and quantitative traits in common buckwheat.

### Detection and origin of novel dominant and recessive QTLs for PHS tolerance

We previously analyzed the segregation of PHS tolerance in F_2_ populations derived from KY29 × KSC7 and KY28 × KSC7 and identified different modes of inheritance between KY29 and KY28 [8]. As we expected, KY29 and KY28 have different QTLs that provide PHS tolerance (Fig. 3, Table 2). QTL analysis and association analysis suggested that the PHS tolerance in KY29 is controlled by dominant alleles at *qHS6-KY29* and partially dominant alleles at *qHS1-KY29*, fitting our expectation [8]. As KY29 was developed from a cross between ‘Kanoya-Zairai’ and ‘Hitachiakisoba’, its PHS tolerance alleles might originate from ‘Kanoya-Zairai’, a Japanese landrace with high tolerance [24].

On the other hand, the PHS tolerance in KY28 is controlled by recessive or partially dominant alleles at *qPHS3_KY28* and *qPHS4_KY28* (Tables 2 and 3). In crosses B_1 and C, two major QTLs were detected on LG3 (*qPHS3_KY28* and *qPHS3_NF1*) and LG8 (*qPHS8_KY28* and *qPHS8_NF1*). KY28 was developed from PHS-tolerant ‘Harunoibuki’ [9] × ‘Hitachiakisoba’, and NF1 was developed from composite crosses among ‘Kitawasesoba’ [25], ‘Yaita-Zairai’, ‘Asahimura-Zairai 3’, ‘Hashikamiwase’, ‘Hitachiakisoba’, ‘Chushinkei VII’, ‘Kyukei 30’, and ‘Kyukei 10’. ‘Harunoibuki’ was developed by the mass selection of low-PHS individuals of ‘Hashikamiwase’ [9]. So the PHS tolerance alleles of *qPHS3_KY28* and *qPHS3_NF1* might originate from ‘Hashikamiwase’.

We detected QTLs which provide PHS tolerance with the KSC7 genotype in three crosses (*qPHS7_KY29*, *qPHS1_KY28*, *qPHS5_KY28, qPHS8_KY28*, and *qPHS8_NF1*; Table 2). KSC7 have been developed from ‘Norin-PL1’ [26] produced from *F. esculentum* × *F. homotropicum*; the latter species has strong seed dormancy [27]. However, we selected lines with a low germination rate to avoid strong seed dormancy for acceleration of the generations during the development of KSC7. Genetic regions contributing strongly to low germination rate would have been eliminated during breeding, but some weak PHS tolerance genes might remain. These QTLs might be useful to improve PHS tolerance. Furthermore, to find new PHS tolerance alleles in *F. homotropicum*, it may be useful to develop lines with strong PHS tolerance, although undesirable traits such as shattering habit would have to be removed.

### Use of PHS tolerance genes in breeding programs

Extensive work has identified genes or major dominant and recessive alleles for PHS tolerance in many plant species, such as wheat, barley, rice, sorghum, and Arabidopsis [28–35]. For example, *QPhs.ocs-3A.1* (identified as *MOTHER-OF-FT-AND-TFL1*) in wheat, *Sdr4* (*Os07g0585700*) in rice, and *DELAY OF GERMINATION1* (*AT5G45830*) in Arabidopsis are dominant or semi-dominant [28–30]. On the other hand, *Qsd1* (*alanine aminotransferase*) in barley, *Qsd2-AK* (*mitogen-activated protein kinase kinase kinase*: *MKK3*) in barley, and *Phs1* (*MKK3*) in wheat are recessive [31, 36, 37]. In wheat and barley, *QPhs.ocs-3A.1*, *Qsd1*, *Qsd2-AK*, and *Phs1* are available for MAS [34].

Using BGDB, we investigated whether there are candidate genes near the QTLs that we identified (Additional files 8 and 9: Tables S8, S9). All scaffolds where QTLs were located contained several open reading frames (ORFs), except Fes_sc0026859.1 (Additional file 8: Table S8). Some ORFs were annotated by BGDB (Additional file 9: Table S9), but no candidate gene seemed to be directly related to PHS. However, because we could not develop markers for all of the scaffolds in this experiment, other scaffolds may exist in the QTL vicinity. Further study will be needed to identify genes controlling each QTL and their function.

Because common buckwheat is an SI plant, dominant alleles are more useful for selecting favorable traits. On the other hand, recessive alleles also can be useful if the favorable genotype can be fixed efficiently by MAS. For example, marker qPHS3_KY28_WG_1572 may be able to select PHS-tolerant progeny efficiently so as to select the homozygous KY28 allele (Table 3). That means that pyramiding by MAS may be useful. This information and markers will be useful for accelerating genetic breeding to improve PHS tolerance in buckwheat.

## Conclusions

We efficiently constructed linkage maps with AmpliSeq technology and performed QTL analysis for PHS tolerance in combination with NGS-BSA in common buckwheat. Candidate markers linked to PHS developed by NGS-BSA were located near these QTL regions. Furthermore, all candidate markers linked to the single genetic locus *S*^*h*^ developed by NGS-BSA were also mapped to near that locus. Thus, we have shown that the combination of NGS-BSA with AmpliSeq is an efficient way to identify genetic regions for both qualitative and quantitative traits in common buckwheat. The QTLs we detected here possess tolerance alleles with different genetic modes: dominant, partially dominant, and recessive. Those alleles could be efficiently selected for in breeding programs by MAS with the STS markers. This is the first report to identify QTLs for PHS tolerance in buckwheat. Our marker development system will accelerate genetic research and breeding in common buckwheat.

## Methods

### Plant materials

Buckwheat is a heteromorphic SI species with two types of floral architecture: thrum (short style) and pin (long style) [38]. This SI system is controlled by a single genetic locus, *S*; thrum is heterozygous (*Ss*) and pin is homozygous recessive (*ss*). We developed SC buckwheat lines from an interspecific cross between common buckwheat, *F. esculentum*, and a self-compatible wild relative, *F. homotropicum* [10]. The SC line has a long homostyle (LH) controlled by a single allele, *S*^*h*^, in the dominance relationship *S* > *S*^*h*^ > *s* [39]. For the development of segregating populations, we used the SC line ‘Kyukei SC7’ (KSC7), which was developed by our research group from the SC line ‘Norin-PL1’ [26].

We used three PHS-tolerant cultivars/breeding lines: ‘Kyukei 29’ (KY29), ‘Kyukei 28’ (KY28), and ‘NARO-FE-1’ (NF1); those were developed by our research group. KY29 and KY28 had significantly higher PHS tolerance than many leading cultivars in Japan and KSC7, and the evaluation is stable among them [5, 8, 24].

We developed five segregating populations (Additional file 1: Table S1) from independent crosses between five PHS-tolerant lines (KY29_1, KY29_2, KY28_1, KY28_2, and NF1) and KSC7. The segregation patterns of the PHS tolerance of crosses A_1 (KY29_1 × KSC7), A_2 (KY29_2 × KSC7), and B_2 (KY28_2 × KSC7) are reported in Hara et al. [8], and those of crosses B_1 (KY28_1 × KSC7) and C (NF1 × KSC7) are newly reported here. For linkage map construction and QTL analysis, segregating F_2_ populations derived from A_1, B_1, and C were used. For association testing of the nearest markers to each QTL, segregating F_2_ populations derived from A_1, A_2, B_1, and B_2 were used (Additional file 1: Table S1).

### Evaluation of PHS tolerance

The F_2_ populations of crosses A_1 (*n* = 132), A_2 (*n* = 106), B_1 (*n* = 100), B_2 (*n* = 106), and C (*n* = 93) and each parental line were grown in a field of the Institute of Crop Science, NARO, Tsukuba, Japan, in 2016 (KY28, NF1, KSC7, A_1, A_2) and 2017 (KY28, KY29, NF1, B_1, B_2, C). Because PHS tolerance evaluated in the field is highly correlated with that evaluated in a Petri dish [8] and the value is stable among lines, we tested PHS tolerance in Petri dishes as described [8]. In brief, 20 freshly collected seeds of each plant were immediately placed on filter paper saturated with distilled water in a Petri dish. The dishes were incubated in a germination cabinet at a constant 25 °C in the dark. Dishes were checked once a day for 7 days, and germinating seeds were counted and removed. Results are percentages. The significance of the differences among parental lines was tested with Tukey–Kramer’s test using the multcomp R package (Additional file 2: Table S2).

### Marker development covering whole genome for AmpliSeq analysis

Genomic DNA was isolated from young leaves of each plant with a DNeasy Plant Mini Kit (Qiagen, Hilden, Germany). We developed genome-wide markers covering the whole genome, PHS-related markers (next section), and SC-related markers (next section). To develop genome-wide markers, we used the sequence information of the markers developed by Yabe et al. [17] on a high-density linkage map of DNA microarrays. We performed local BLAST searches of all microarray probe sequences used for constructing the linkage map as queries for the BGDB reference genome (FES_r1.0). The searches were performed by Galaxy BLASTN v. 2.7.1 with the default setting. After determining the scaffold number of each microarray probe, we evaluated these scaffolds as genome-wide scaffolds. From these and resequencing data between KY29 and KSC7 (parents of A_1), we selected 300 SNP sites.

### Marker development by NGS-BSA for PHS and SI/SC

To develop PHS-linked markers, we used bulked DNA from 46 highly PHS-tolerant plants (0% PHS) and bulked DNA from 10 plants with low PHS tolerance (> 50% PHS) from the F_2_ population of cross A_1. Paired-end reads of 100 bp from the two bulks were obtained on an Illumina HiSeq-X System at Macrogen Japan (Kyoto, Japan; DDBJ/EMBL/NCBI accession number PRJDB9892). To investigate the effect and certainty of NGS-BSA, we made a set of markers linked to the SC allele *S*^*h*^ from previously published WGS data of KSC7 and KY29 (accession number DRX178921) [13].

Low-quality reads and adaptors (CACGACGCTCTTCCGATCT and ACCGCTCTTCCGATCTGTAA) were trimmed in Trimmomatic v. 0.32 software [40] with settings of HEADCROP 2, SLIDINGWINDOW 4:25, LEADING 25, TRAILING 25, and MINLEN: 40. Trimmed reads were mapped to the reference sequences in BWA v. 0.7.15 software [41] with the ‘bwa aln’ option (−l 32, −k 2, −n 5) and the ‘bwa sample’ option (default settings). Only genomic sequences [11] of ≥1 kb were selected as reference sequences. Mapping results were processed in SAMtools v. 0.1.18 software [42].

SNPs were detected with the UnifiedGenotyper tool in GATK v. 3.7 software [43] with the -glm BOTH option. PHS resistance–linked SNPs were detected with the following criteria: (1) SNPs between KSC7 and KY29 with the depth > 10; (2) heterozygous in high-PHS-tolerance bulked DNA; (3) homozygous with the same nucleotide as KSC7 in low-PHS-tolerance bulked DNA. The reference sequence in the BGDB is divided into 387,594 short scaffolds (N50 = 25.1 kb) [11], and it is difficult to depict changes in the number of high-PHS-tolerance–linked SNP sites through the scaffolds. Hence, we counted those SNPs in all reference sequences and calculated the ratio of the number of PHS-linked SNPs to the number of all SNPs as the “PHS-linked SNP index” for each scaffold. The top 100 markers of candidate genes in PHS and SI/SC were used for mapping. A custom panel targeting 500 regions (300 as the whole genome, 100 as PHS-linked, and 100 as *S*^*h*^ -linked) was designed based on the KSC7 custom reference genome using the Ion AmpliSeq Designer (https://ampliseq.com/help/startDesign.action) [44] version 6.0 using the standard DNA (125–275 bp amplicon target sizes) option.

### Construction of AmpliSeq library, sequencing, variant detection, and genotyping

The AmpliSeq library was prepared with the Ion AmpliSeq Library Kit 2.0 and the IonCode Barcode Adapter 1–384 Kit (both from Thermo Fisher Scientific) as described in Ogiso-Tanaka et al. [15]. Multiplex PCR amplification was performed in a total reaction volume of 5 μL manually or 4.8 μL robotically on a Mosquito HV instrument (TTP Labtech, Royston, UK) [45], with 10 ng of each genomic DNA (*n* = 100 in A and B crosses, *n* = 93 in C cross). The Ion Library Equalizer Kit was used to normalize the library concentration to 100 pM, and libraries were pooled and sequenced on an Ion Torrent S5 system (Thermo Fisher Scientific). Template preparation (emulsion PCR, enrichment of beads containing template, and chip loading) was performed with the Ion Chef instrument and Ion S5 Kit-Chef according to the manufacturer’s instructions. After the preparation of ion sphere particles, sequencing for 500 cycles was performed on an Ion Torrent S5 system using an Ion 540 Chip according to the manufacturer’s instructions. The sequence data were mapped to the KSC7 custom reference genome by the Ion Torrent Mapping Alignment Program v. 5.8.0 in Torrent Suite v. 5.8.0 software. Coverage analysis and variant detection were performed in Coverage Analysis v. 5.8.0.8 and variantCaller v. 5.8.0.19 software with default parameters (Germ Line with low stringency). All detected variants were listed in a hotspot VCF file. Variants at hotspot sites were detected in variantCaller with default parameters. Finally, the genotype file for the R/qtl package (http://www.rqtl.org/) [46] was obtained by converting from the output file of variantCaller in the IonBreeders ABH plugin [47].

### Construction of genetic linkage map and QTL analysis

Before constructing a linkage map, we preprocessed data in R/qtl as follows: (1) Find the duplicate markers which show the same genotyping in all individuals except for missing value, select the markers with the fewest missing values, and drop duplicate markers. (2) Drop samples with < 80 genotypes. (3) Drop marker subsets in which < 90% of the population is genotyped. (4) Drop markers with an abnormal genotype distribution. The genetic map was constructed in AntMap v. 1.2 software [48]. QTL analysis was performed in WinQTL Cartographer v. 2.5 software using the composite interval mapping (CIM) model [49]. The significance threshold of the log-likelihood (LOD) score was based on 1000 permutations (*P* = 0.05).

### Development of STS markers linked to QTLs

According to the resequencing data between KY29 and KSC7 or sequencing data from AmpliSeq, we converted QTL nearest AmpliSeq markers to STS markers. Amplification with genomic DNA as a template was performed with the designed specific primers by *ExTaq* (TaKaRa, Shiga, Japan) as follows: 32 cycles at 94 °C for 30 s, 58 °C for 30 s, and 72 °C for 30 s. Amplification was confirmed by agarose gel electrophoresis, and the DNA fragments were digested with restriction enzymes (*AfaI*, *AluI*, *EcoRI*, or *MspI* (all from TaKaRa)). The primer sequences and the combination of the restriction enzymes are listed in Additional file 7: Table S7.

## Supplementary Information

**Additional file 1: Table S1.** List of plant materials.

**Additional file 2: Table S2.** Ratio of pre-harvest sprouting of the parent lines.

**Additional file 3: Table S3.** Association with array marker sequences (Yabe et al., [17]) and BGDB scaffolds.

**Additional file 4: Table S4.** Selected 300 SNP sites between micro array marker sequences (Yabe et al., [17]) and BGDB scaffolds.

**Additional file 5: Table S5.** Identification of scaffolds linked to PHS tolerance based on SNP index.

**Additional file 6: Table S6.** Summary information for AmpliSeq custom panel.

**Additional file 7: Table S7.** Primer information for sequence-tagged-site markers linked to QTLs.

**Additional file 8: Table S8.** Scaffolds where QTLs are located and closest marker to each QTL.

**Additional file 9: Table S9.** Genes annotated near QTLs.

**Additional file 10: Figure S1.** Box plots of on-target rate and uniformity. On-target rate is shown as percentage of aligned reads. Coverage uniformity score is shown as percentage of area covered at ≥0.2× mean coverage depth. Box plots show median and interquartile range; red, Cross A (*n* = 94); green, Cross B (*n* = 87); blue, Cross C (*n* = 84).

## Data Availability

All data generated or analyzed during this study are included in the manuscript and its Additional files 1–10. The raw reads of next-generation sequencing-based bulked-segregant analysis obtained in this study is available from the DDBJ/EMBL/NCBI under the accession number PRJDB9892 (http://trace.ddbj.nig.ac.jp/BPSearch/bioproject?acc=PRJDB9892). The datasets used and/or analyzed during the current study are available from the corresponding author on reasonable request.

## Abbreviations

BGDB: Buckwheat genome database
BSA: Bulked-segregant analysis
CIM: Composite interval mapping
KSC7: ‘Kyukei SC7’
KY28: ‘Kyukei 28’
KY29: ‘Kyukei 29’
LG: Linkage group
LH: Long homostyle
LOD: Log-likelihood
MAS: Marker-assisted selection
NF1: ‘NARO-FE-1’
NGS: Next-generation sequencing
ORF: Open reading frame
PHS: Preharvest sprouting
QTL: Quantitative trait locus
SC: Self-compatibility
SI: Self-incompatibility
STS: Sequence-tagged-site
WGS: Whole-genome resequencing
