# Supplementary material for: Targeted amplicon sequencing + next-generation sequencing–based bulked segregant analysis identified genetic loci associated with preharvest sprouting tolerance in common buckwheat (Fagopyrum esculentum)
Source: BMC Plant Biol. 2021 Jan 6;21:18. doi: 10.1186/s12870-020-02790-w (PMC7789488; doi:10.1186/s12870-020-02790-w)
Supplement: Supplementary file 10 — Additional file 10: Figure S1. Box plots of on-target rate and uniformity. On-target rate is shown as percentage of aligned reads. Coverage uniformity score is shown as percentage of area covered at ≥0.2× mean coverage depth. Box plots show median and interquartile range; red, Cross A (n = 94); green, Cross B (n = 87); blue, Cross C (n = 84). [file 12870_2020_2790_MOESM10_ESM.pptx]

## Slide 1
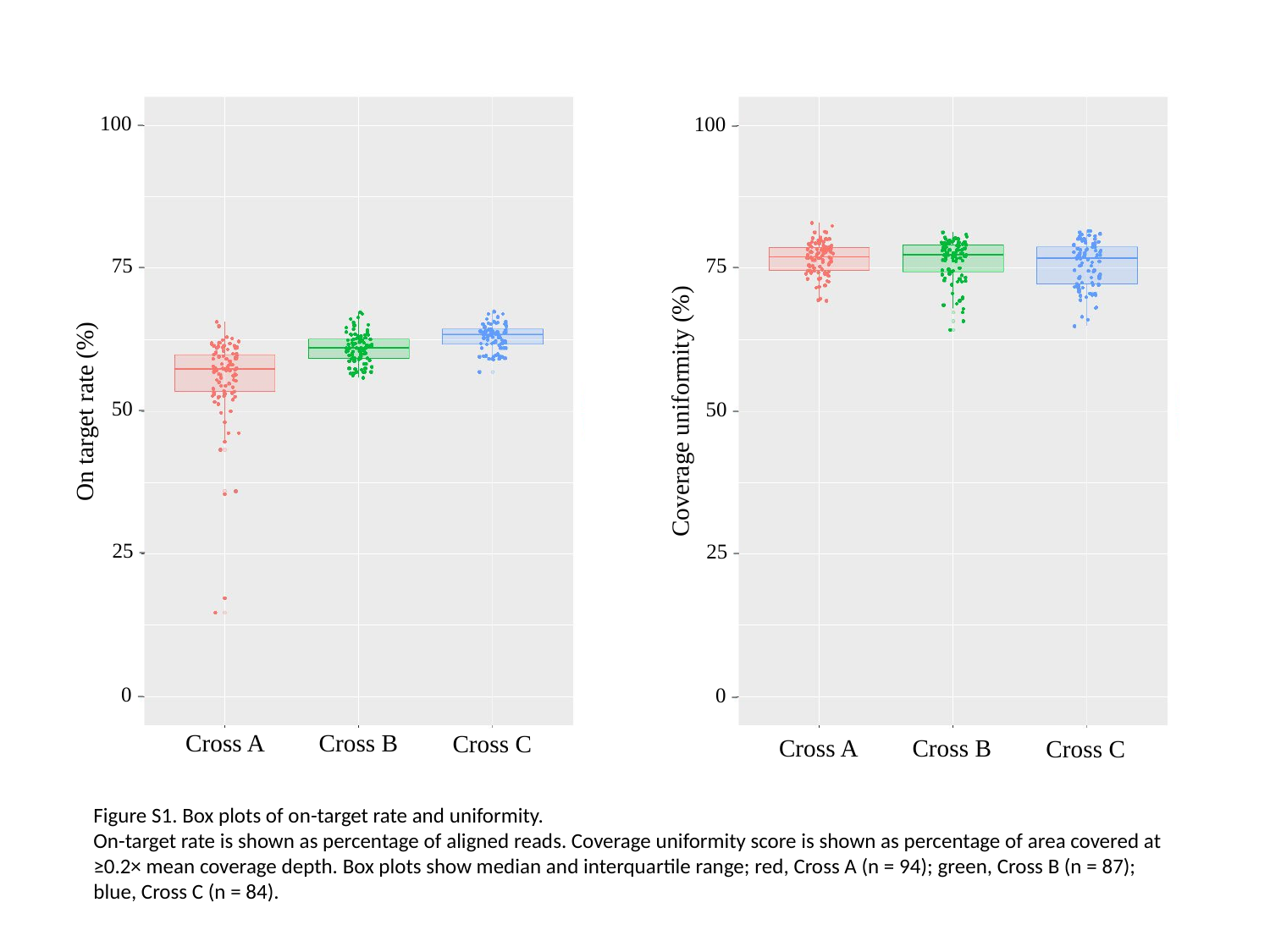

100 -
75 -
50 -
25 -
0 -
100 -
75 -
50 -
25 -
0 -
On target rate (%)
Coverage uniformity (%)
Cross A
Cross B
Cross C
Cross A
Cross B
Cross C
Figure S1. Box plots of on-target rate and uniformity.
On-target rate is shown as percentage of aligned reads. Coverage uniformity score is shown as percentage of area covered at ≥0.2× mean coverage depth. Box plots show median and interquartile range; red, Cross A (n = 94); green, Cross B (n = 87); blue, Cross C (n = 84).
